# Nimbolide Represses the Proliferation, Migration, and Invasion of Bladder Carcinoma Cells via Chk2-Mediated G2/M Phase Cell Cycle Arrest, Altered Signaling Pathways, and Reduced Transcription Factors-Associated MMP-9 Expression

**DOI:** 10.1155/2019/3753587

**Published:** 2019-07-14

**Authors:** Seung-Shick Shin, Byungdoo Hwang, Kashif Muhammad, Yujeong Gho, Jun-Hui Song, Wun-Jae Kim, Gonhyung Kim, Sung-Kwon Moon

**Affiliations:** ^1^Department of Food Science and Nutrition, Jeju National University, Jeju 63243, Republic of Korea; ^2^Department of Food and Nutrition, Chung-Ang University, Anseong 17546, Republic of Korea; ^3^College of Veterinary Medicine, Chungbuk National University, Cheongju, Chungbuk 361-763, Republic of Korea; ^4^Department of Urology, Chungbuk National University, Cheongju, Chungbuk 361-763, Republic of Korea

## Abstract

Nimbolide, an active chemical constituent of* Azadirachta indica*, reportedly has several physiological effects. Here, we assessed novel anticancer effects of nimbolide against bladder cancer EJ and 5637 cells. Nimbolide treatment inhibited the proliferation of both bladder cancer cell lines with an IC_50_ value of 3 *μ*M. Treatment of cells with nimbolide induced G2/M phase cell cycle arrest via both Chk2-Cdc25C-Cdc2/cyclin B1-Wee1 pathway and Chk2-p21WAF1-Cdc2/cyclin B1-Wee1 pathway. Nimbolide increased JNK phosphorylation and decreased p38MAPK and AKT phosphorylation. Additionally, nimbolide impeded both wound healing migration and invasion abilities by suppressing matrix metalloproteinase-9 (MMP-9) activity. Finally, nimbolide repressed the binding activity of NF-*κ*B, Sp-1, and AP-1 motifs, which are key transcription factors for MMP-9 activity regulation. Overall, our study indicates that nimbolide is a potential chemotherapeutic agent for bladder cancer.

## 1. Introduction

Bladder cancer is the 10th most common malignancy worldwide, with an incidence and mortality of 9.6 and 3.2 per 100,000 in men, respectively [1], and a 4-fold greater incidence in men than in women [1]. The development and progression of bladder cancer are associated with multiple factors such as environmental conditions, reactive oxygen species, chemical carcinogens, and anticancer drugs [2]. The critical progressive type of bladder tumors is a muscle invasive bladder cancer (also known as transitional cell carcinoma) that typically occurs in the urinary system and possesses proliferative, migratory, and invasive potentials [3]. Although modalities involving hormone therapy, chemotherapy, surgery, and radiotherapy are available for treating bladder cancer, issues concerning economic costs and adverse toxic side-effects associated with these modalities remain unsolved [4]. Therefore, the development of a novel therapeutic agent that is inexpensive and has minimal side-effects is urgently warranted.

The progression of bladder cancer arising from urothelial carcinoma involves a cascade signaling process implicated in the phosphorylation of early signaling molecules such as mitogen-activated protein kinases (MAPKs) and PI3K/AKT [5–7]. Uncontrolled proliferation of cancer cells occurs owing to cell cycle progression at the G_1_-, S-, and G_2_/M cell-cycle phases [8, 9]. Regulatory proteins associated with G2/M phase progression, including cyclin B, cdc2, cdc25c, p21WAF1, Wee1, and p27KIP1, have recently attracted attention as the primitive targets of chemotherapeutic reagents [10]. The migration and invasion of cancer cells derived by proteinase, matrix metalloproteinases (MMPs), are an equally promising target for bladder cancer [3, 11]. MMP-9 activity is closely linked with migratory and invasive potential and correlates with the occurrence and progression of bladder cancer [3, 12, 13]. During migration and invasion of bladder cancer cells, MMP-9 regulation is controlled via activation of the transcription factors NF-*κ*B, Sp-1, and AP-1 [11, 14, 15].

*Azadirachta indica*, commonly known as neem, is a traditional medicinal plant of the Meliaceae family and distributed in Asia, Africa, and other tropical regions of the world. In many countries, the flowers and leaves of neem trees are widely consumed by people and animals as traditional medicine to heal various ailments and diseases [16]. The leaf extract of neem has been suggested to be nonmutagenic and nontoxic [17]. Nimbolide, a limonoid derivative from the seeds and leaves of neem tree, is a tetranortriterpenoid containing an *α*,*β*-unsaturated system and a *δ*-lactonic ring [18]. It reportedly exerts various physiological activities including insecticidal, antifeedant, antimalarial, and anticancer activities [19, 20]. Although it demonstrated antiproliferative activity against many kinds of cancer cells, including those of lung cancer, breast cancer, osteosarcoma, neuroblastoma, melanoma, and choriocarcinoma [18, 21], information regarding the molecular mechanisms of nimbolide-mediated anticancer effects are largely obscure. In this study, we examined the molecular signaling cascades of nimbolide-induced suppression of proliferation, migration, and invasion of bladder cancer EJ cells by conducting an inclusive analysis of signaling pathway, cell cycle modulation, and transcription factor-controlled MMP-9 regulation.

## 2. Materials and Methods

### 2.1. Materials

Nimbolide was purchased from Sigma-Aldrich (SMB00586, St. Louis, MO, USA). Nimbolide was dissolved in dimethyl sulfoxide (DMSO). Antibodies against phospho-Cdc25C (Ser 216) (sc-12354), Cdc25C (sc-327), phospho-Cdc2 (Tyr 15) (sc-12340-R), Cdc2 (sc-54), cyclin B1 (sc-245), Weel (sc-325), p21WAF1 (sc-756), p27KIP1 (sc-528), p53 (sc126), and GAPDH (sc-47724) were purchased from Santa Cruz Biotechnology (Dallas, TX, USA). Antibodies against checkpoint kinase (Chk)1 (#2360), Chk2 (#2662), phospho-Chk1 (Ser345) (#2341), phospho-Chk2 (Thr68) (#2661), ERK (#9102), p38MAPK (#9212), JNK (#9258), AKT (#9272), phospho-ERK (#9101), phospho-p38MAPK (#9211), phospho-JNK (#9251), and phospho-AKT (#9271) were purchased from Cell Signaling Technology (Danvers, MA, USA). The nuclear extract kit and the* electrophoretic mobility shift assay* (EMSA) gel shift kit (AY1XXX) were obtained from Panomics (Fremont, CA, USA).

### 2.2. Cell Culture

The human bladder carcinoma cell lines (EJ and 5637) were provided by Dr. Wun-Jae Kim (Department of Urology, Chungbuk National University, Chungbuk, South Korea). Both cell lines were grown in Dulbecco's modified Eagle's medium containing 10% fetal bovine serum (FBS), 100 U/mL penicillin, and 100 *μ*g/mL streptomycin at 37°C in a 5% CO_2_ humidified incubator, although the EJ cell line is known to be cross-contaminated with the T24 cells, which were also derived from a bladder carcinoma [22, 23]. Therefore, this contamination issue was not expected to affect the results of this study.

### 2.3. Nimbolide Treatment and MTT Assay

Both cell lines (5 × 10^3^ cells/well) were seeded in 96-well plates and then treated with nimbolide (0, 0.5, 1, and 3 *μ*M) for 12 h. Subsequently, cell viability was checked for varying concentrations of nimbolide using MTT (the 3-(4,5-dimethylthiazol-2-yl)-2,5-diphenyltetrazolium bromide) assay. Briefly, cells (5 × 10^3^/well) were cultured in 96-well plates and incubated with nimbolide for 12 h. After removing the medium, fresh medium supplemented with 10 *μ*L of 5 mg/mL MTT was added for 1 h. The medium was removed and 100 *μ*L of dimethyl sulfoxide (DMSO) was added. Absorbance at 540 nm was evaluated using a microplate reader.

### 2.4. Viable Cell Counting

Both cell lines (3 × 10^5^ cells/well) were cultured and treated with nimbolide (0, 0.5, 1, and 3 *μ*M) in 6-well plates for 12 h. The cells were then detached by adding 0.25% trypsin containing 0.2% EDTA (ThermoFisher Scientific, Waltham, MA, USA) and mixed with 50 *μ*L of 0.4% trypan blue (Sigma-Aldrich) via gentle pipetting. Finally, after adding the mixture (20 *μ*L) into each chamber of a hemocytometer, stained cells were counted. The morphology of the cells was observed using a phase-contrast microscope and photographed.

### 2.5. Cell Cycle Analysis

Both cell lines were treated with nimbolide and then detached with trypsin. Collected cells were fixed with 70% ethanol. After washing with ice-cold phosphate buffered saline (PBS), harvested cells were reacted with RNase (1 mg/mL) and propidium iodide (50 mg/mL). The cell cycle distribution was assessed using a flow cytometer (FACStar, BD Biosciences, San Jose, CA) accompanied with BD Cell Fit software.

### 2.6. Immunoblots

Cells (3 × 10^5^ cells/well) were seeded in 6-well plates and then treated with nimbolide (0, 0.5, 1, and 3 *μ*M) for 12 h. Cultured cells were then washed with 1× cold PBS twice and then freeze-thawed in 200 *μ*L lysis buffer (composition: 50 mM HEPES, pH 7.5, 150 mM NaCl, 1 mM EDTA, 1 mM DTT, 2.5 mM EGTA, 10 mM *β*-glycerophosphate, 0.1 mM Na_3_VO_4_, 1 mM NaF, 0.1 mM PMSF, 10% glycerol, 0.1% Tween-20, 10 *μ*g/mL leupeptin, and 2 *μ*g/mL aprotinin). After the cells were scraped into 1.5-mL Eppendorf tubes, the collected cells were incubated in ice for 10 min. The cell lysates were subsequently obtained by centrifuging at 10,000 ×* g* for 10 min at 4°C. The total amount of protein in the supernatant was evaluated using a BCA protein assay reagent kit (Thermo Fisher Scientific). For each sample, lysates containing 25 *μ*g of protein were subjected to a 10% polyacrylamide gel (SDS-PAGE) while maintaining denaturing conditions. After electrophoresis, proteins were transferred onto nitrocellulose membranes (Hybond, GE Healthcare Bio-Sciences, Marlborough, MA, USA). Nonspecific binding of proteins with the membrane was blocked by reaction with 5% skimmed milk. After the membrane was washed with PBS, it was incubated overnight with primary antibodies. The membrane was further incubated with a peroxidase-conjugated secondary antibody for 90 min. The immunocomplexes were detected using a chemiluminescence reagent kit (GE Healthcare Lifesciences, Marlborough, MA, USA). All experiments were repeated at least three times. GAPDH and nonphosphorylated forms of antibodies were used as internal control.

### 2.7. Wound-Healing Migration Assay

Cells under the exponential growth phase were grown in 6-well plates (3 × 10^5^/well). To eliminate migration induced by cell proliferation, the cells were preincubated with 5 *μ*g/mL mitomycin C (Sigma-Aldrich) for 2 h. An allocated area of the cell surface was scratched with a wide pipette tip (2 mm). After the plates were washed with PBS buffer, nimbolide was added (0, 0.5, 1, and 3 *μ*M) and subsequently incubated for 12 h. The potential of the cells to migrate into the wound area was evaluated and compared with that of the control cells. Morphological changes of the cells in response to nimbolide treatment were captured using an inverted microscope at 40× magnification.

### 2.8. Boyden Chamber Invasion Assay

The invasive ability of the cells (2.5 × 10^4^) was evaluated in the upper chamber of a Matrigel-coated Transwell plates with 8-*μ*m pores (Sigma-Aldrich). Briefly, cells were resuspended in FBS-free media supplemented with mitocycin C (5 *μ*g/mL). After incubation for 2 h, the cells were seeded in the upper chamber, and nimbolide was added (0, 0.5, 1, and 3 *μ*M) for 12 h. Then, growth medium containing 10% FBS was loaded into the lower chamber as a chemoattractant for 24 h. The cells that had invaded into the lower chamber were fixed, stained, and photographed.

### 2.9. Zymography

Cells were plated and treated with different concentrations of nimbolide for 12 h. Then, the conditioned medium was obtained and subjected to electrophoresis on a polyacrylamide gel containing 0.25% gelatin. The gel was rinsed twice for 15 min at room temperature using 2.5% Triton X-100. The gel was continuously incubated in a reaction buffer (composition: 150 mM NaCl, 50 mM Tris-HCl, and 10 mM CaCl_2_; pH 7.5) at 37°C overnight. The gel was stained with 0.2% Coomassie Blue and destained with a solution containing 10% acetic acid and 10% methanol in distilled water. Nonstaining bands were photographed on a light box. Gelatinase activity was represented as a white zone of gelatin digestion in a dark-blue field. The levels of MMP-9 activity were assessed via densitometric measurements.

### 2.10. Nuclear Extracts and Electrophoretic Mobility Shift Assay (EMSA)

Both cell lines were treated with various concentrations of nimbolide (0, 0.5, 1, and 3 *μ*M) for 12 h. Nuclear extracts were isolated using a Nuclear Extraction kit (Panomics). Briefly, the cell pellets were collected by centrifugation, washed, and resuspended in a buffer comprising 10 mM HEPES (pH 7.9), 10 mM KCl, 1 mM DTT, 0.5 mM PMSF, 0.1 mM EDTA, and 0.1 mM EGTA. Before the cells were lysed with 0.5% NP-40, cellular pellets were maintained in ice for 15 min. Nuclear extracts were collected by centrifugation followed by extraction using an ice-cold high salt buffer [20 mM HEPES (pH 7.9), 400 mM NaCl, 1 mM DTT, 1 mM PMSF, 1 mM EDTA, and 1 mM EGTA] at 4°C for 15 min. After centrifugation, the supernatant containing the nuclear extract was acquired. The total amount of nuclear protein was quantified using a BCA protein assay reagent kit (Thermo Fisher Scientific). The nuclear extract (20 *μ*g) was preincubated at 4°C for 30 min with a 100-fold excess of unlabeled oligonucleotide probes containing −79 position of the MMP-9 cis-acting element of interest. The oligonucleotide sequences were as follows: AP-1, CTGACCCCTGAGTCAGCACTT; NF-*κ*B, CAGTGGAATTCCCCAGCC; and Sp-1, GCCCATTCCTTCCGCCCCCAGATGAAGCAG. The reaction mixture was serially incubated in a buffer (25 mM HEPES, pH 7.9), 0.5 mM EDTA, 0.5 mM DTT, 50 mM NaCl, and 2.5% glycerol) at 4°C for 20 min using 2 *μ*g poly dI/dC and 5 fmol (2 × 10^4^ cpm) of Klenow end-labelled (^32^P ATP) 30-mer oligonucleotide that consists of the DNA-binding motif of the MMP-9 promoter. Subsequently, the reaction mixture was separated by electrophoresis on a 6% polyacrylamide gel at 4°C, and the gel was exposed to X-ray film overnight. The density of the blots was evaluated using the ImagePro Plus 6.0 software (Media Cybernetics, Rockville, MD, USA).

### 2.11. Statistical Analysis

Where appropriate, data were represented as the mean ± the standard deviation (SD) and were analyzed using factorial analysis of variance (ANOVA) and Fisher's least significant difference test. Values of* p* < 0.05 were considered statistically significant.

## 3. Results

### 3.1. Nimbolide Inhibits the Proliferation of EJ and 5637 Cells

**Figure 1:**
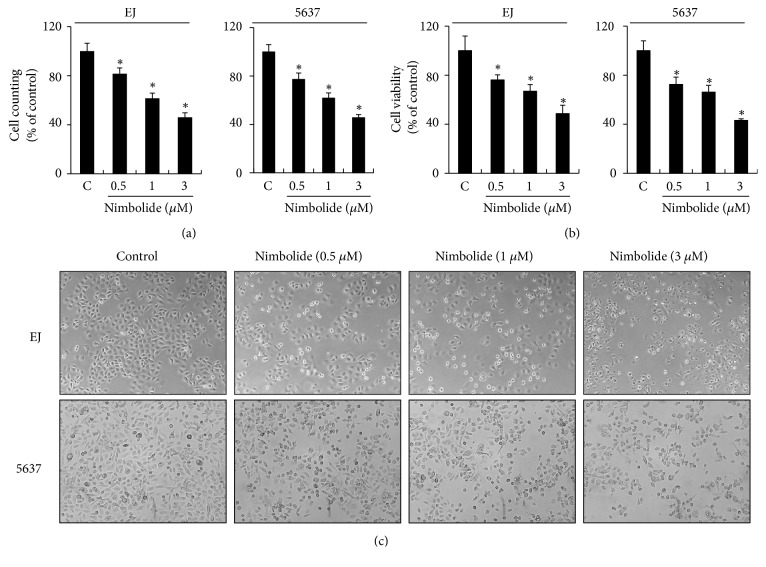
Nimbolide suppresses the proliferation of bladder cancer EJ and 5637 cells. (a) Both cell lines were treated with various concentrations of nimbolide for 12 h, followed by cell counting assay. (b) MTT assay was performed to evaluate cell viability. (c) Cellular morphology was viewed under a phase contrast microscope at 40** ×**. Values in the bar graphs represent the mean ± standard deviation (SD) of three independent experiments; *∗P *< 0.05, compared with the control group.

To examine the proliferation of EJ cells, cell counting and MTT assays were performed. Present results revealed that the number of nimbolide-treated EJ cells and their cell viability reduced in a concentration-dependent pattern (Figures 1(a) and 1(b), respectively) in comparison with those of untreated cells. Similar results were observed in the cell counting and MTT assays in 5637 cells (Figures 1(a) and 1(b)). The IC_50_ value was found to be approximately 3 *μ*M in both EJ and 5637 cells. In addition, morphological changes were monitored using phase contrast microscope. Both nimbolide-treated cell lines exhibited marked changes, such as condensed cell shape and decreased cell density, as opposed to untreated cells (Figure 1(c)). These results demonstrated that nimbolide inhibited the proliferation of EJ and 5637 cells in a dose-dependent manner.

### 3.2. Nimbolide Stimulates G2/M Phase Cell Cycle Arrest in Bladder Cancer Cells

**Figure 2:**
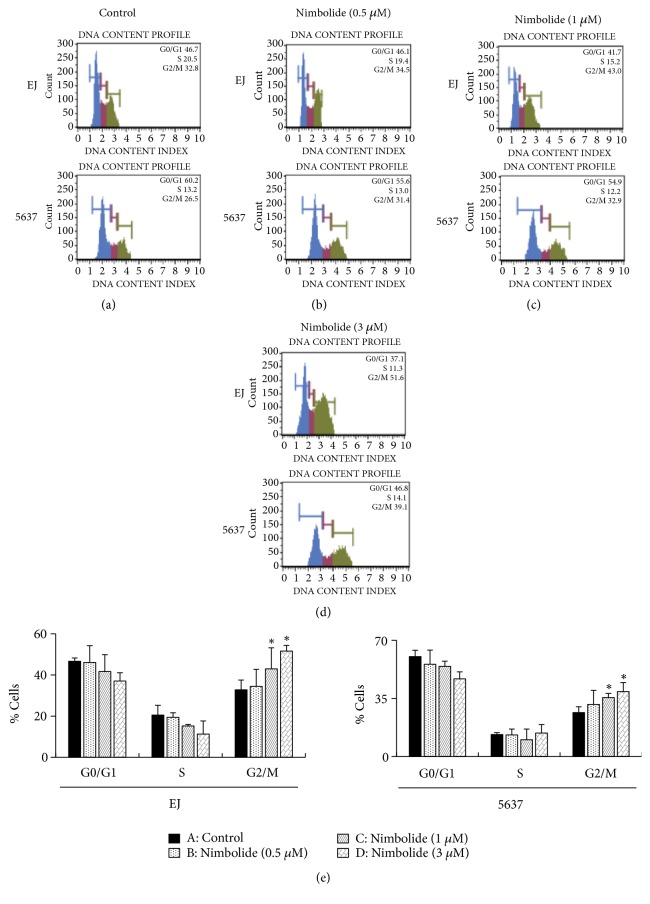
Nimbolide induces G2/M phase cell cycle arrest in bladder cancer cells. Flow cytometry analysis was performed to investigate the cell cycle distribution of the cells treated with 0 (a), 0.5 (b), 1 (c), and 3 (d) *μ*M nimbolide for 12 h. (e) The percentage of cells in G1-, S-, and G2/M phases was analyzed by treatment with varying concentrations of nimbolide for 12 h. Values in the bar graph represent mean ± SD of three independent experiments: *∗P *< 0.05, compared with the control group.

To investigate the alteration caused in the cell cycle, EJ and 5637 cells were treated with different concentrations of nimbolide for 12 h. Cell cycle distribution was investigated using flow cytometry. The results of cell cycle analysis revealed that nimbolide significantly induced an accumulated proportion of cells in the G2/M phase (Figures 2(a)–2(e)). In addition, nimbolide treatment decreased the percentage of cells in both G1- and S phases (Figures 2(a)–2(e)). These results suggested that nimbolide caused G2/M phase cell cycle arrest in EJ and 5637 cells.

### 3.3. Nimbolide Alters the Level of G2/M Phase Cell Cycle Regulatory Proteins in Bladder Cancer Cells

**Figure 3:**
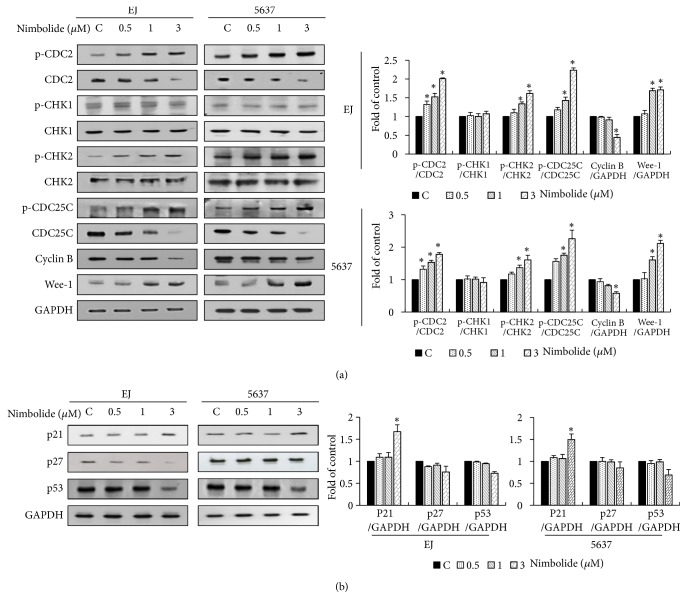
Changes in the cell cycle regulators in the G2/M phase induced by nimbolide in bladder cancer cells. Both cell lines were treated with nimbolide for 12 h, and protein lysates were analyzed by immunoblotting. (a) The expression level of G2/M phase-associated cell cycle regulators were assessed using various kinds of specific antibodies. GAPDH was used for the normalization of protein levels. (b) Negative cell cycle regulators were analyzed via immunoblotting by employing specific antibodies against p21WAF1, p27KIP1, and p53. Bar graphs show the relative ratios of each protein level represented as a fold change in comparison with the control. Each value was expressed as the mean ± SD of three independent experiments; *∗P *< 0.05, compared with the control group.

To better understand the cell cycle-associated mechanism of nimbolide, we examined the level of the major regulatory proteins that control progression of the G2/M cell cycle phase. In both EJ and 5637 cells, nimbolide treatment increased the degree of Chk2 phosphorylation without affecting the level of Chk2 proteins (Figure 3(a)). In contrast, the phosphorylation and protein level of Chk1 was not changed by nimbolide addition (Figure 3(a)). Moreover, nimbolide treatment increased Cdc25c phosphorylation but decreased Cdc25c protein level in bladder cancer cells (Figure 3(a)). Furthermore, an increased phosphorylation of Cdc2 was also observed in the presence of nimbolide (Figure 3(a)). Treatment with nimbolide decreased protein level of Cdc2 and cyclin B1 (Figure 3(a)). Finally, nimbolide treatment enhanced both p21WAF1 and Wee1 expression (Figures 3(a) and 3(b)). However, the expression level of both p27KIP1 and p53 was slightly decreased in the presence of nimbolide (Figure 3(b)). The results of the present study indicated that nimbolide induced the G2/M phase cell cycle arrest in bladder cancer cells via Chk2-Cdc25c-Cdc2/cyclin B1-Wee1 axis and Chk2-p21WAF1-Cdc2/cyclin B1-Wee1 axis.

### 3.4. Nimbolide Induces JNK Phosphorylation and Inhibits p38MAPK and AKT Phosphorylation in Bladder Cancer Cells

**Figure 4:**
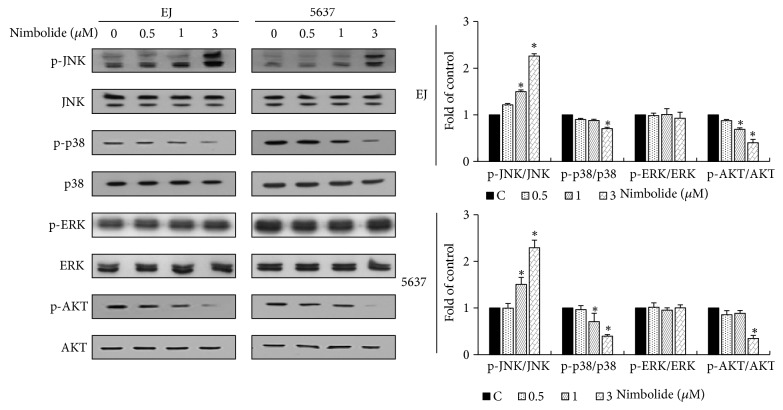
Nimbolide induces JNK phosphorylation and inhibits p38MAPK and AKT phosphorylation in bladder cancer cells. Both EJ and 5637 cells were treated with the indicated concentrations of nimbolide for 12 h. Cell lysates were prepared and the expression levels of proteins were determined via immunoblotting by employing specific antibodies against ERK1/2, p38MAPK, JNK, and AKT. The relative level of the phosphorylated to the unphosphorylated form was evaluated and represented as a fold change in comparison with the control. Each value was expressed as the mean ± SD of three independent experiments; *∗P *< 0.05, compared with the control group.

It has been recognized that bladder cancer progression involves the phosphorylation of early signaling pathway molecules, such as the MAPKs (ERK, JNK, and p38MAPK) and AKT [5–7]. Hence, we aimed to investigate whether nimbolide affected the expression level of the phosphorylated form of MAPKs and AKT. Immunoblot results revealed that nimbolide treatment induced JNK phosphorylation in EJ and 5637 cells (Figure 4). In contrast, the levels of phosphorylated p38MAPK and AKT decreased but ERK phosphorylation remained unchanged in both nimbolide-treated bladder cancer cell lines (Figure 4). These results suggest that nimbolide-induced inhibition of cell growth is involved in the upregulation of JNK phosphorylation and downregulation of p38MAPK and AKT phosphorylation in EJ and 5637 cells.

### 3.5. Nimbolide Inhibits the Migration and Invasion of Bladder Cancer Cells

**Figure 5:**
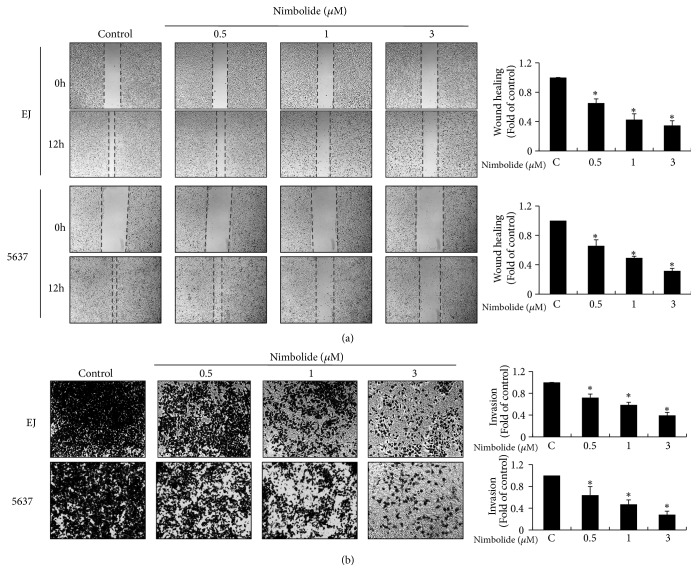
The suppressive effect of nimbolide in the migration and invasion of bladder cancer cells. (a) Both cell lines were grown in 6-well plates. Monolayer of cells were scratched using a pipette tip. After treatment with nimbolide (0–3 *μ*M) for 12 h, the recovery rate of cells migrating into the wound surface was monitored and evaluated as the width of the remaining wounded area relative to the initial wounded area. Wound closure images were photographed under an inverted microscope with 40× magnification. (b) Invasiveness of both bladder cancer cell lines was assessed using an invasion assay kit. Cells were plated in the upper Boyden chamber, and then cells that had invaded to the lower chamber were stained. Cellular images were visualized under a microscope after 12 h. The bar graphs indicate the number of migrating or invading cells expressed as a fold change relative to the control. Values in the bar graphs represent the mean ± SD from three different experiments. *∗P *< 0.05, compared to the control.

To clarify the migratory and invasive potential of nimbolide, wound-healing migration and invasion assay was performed in both EJ and 5637 cells. The ability of wound-healing migration was determined by movement of cells into the wound area. Nimbolide treatment suppressed the migration of cells into the wounded area as compared to the untreated cells (Figure 5(a)). Next, the invasive potential was evaluated using Matrigel®-coated transwell plates. As shown in Figure 5(b), the number of nimbolide-treated cells that invade through the transwell basement membrane was lower than that treated with the medium alone. These results show that nimbolide impedes the migration and invasion of EJ and 5637 cells.

### 3.6. Nimbolide Suppresses MMP-9 Activity via Repression of the Transcription Factors NF-*κ*B, AP-1, and Sp-1 in Bladder Cancer Cells

**Figure 6:**
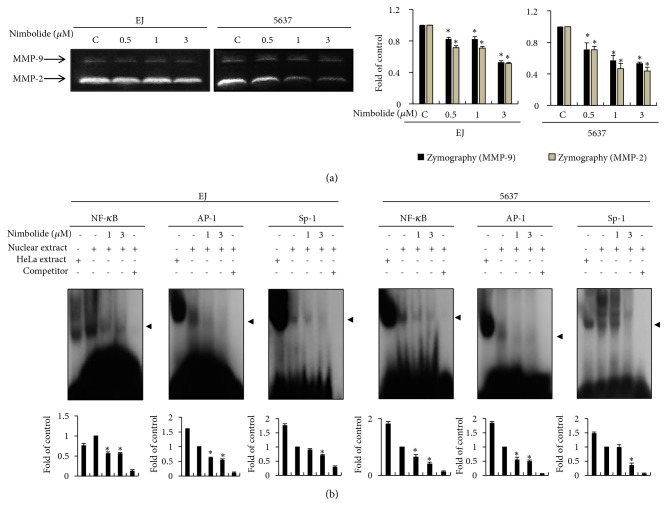
Nimbolide inhibits MMP-9 activity via interfered binding activity of transcription factors NF-*κ*B, AP-1, and Sp-1 in bladder cancer cells. (a) MMP-2 and MMP-9 activities were measured by gelatin zymographic assay. Both cell lines were grown in a petri dish and incubated with various concentrations of nimbolide. Zymographic assay was performed using conditioned media. (b) Nuclear proteins were used by EMSA. NF-*κ*B, AP-1, and Sp-1 motifs were employed to analyze transcriptional binding activity using radiolabeled oligonucleotide probes. Bar graph is presented as a fold change relative to the control. The results are presented as the mean ± SD from three different experiments. *∗P *< 0.05, compared to the control.

Activity of MMPs, such as MMP-2 and MMP-9, is essential for the degradation of extracellular matrix (ECM), which results in the migration and invasion of tumor cells [3, 11]. Enzymatic activities of both MMP-2 and MMP-9 were analyzed by gelatin zymography in EJ and 5637 cells. The proteolytic activity of both MMP-2 and MMP-9 in nimbolide-treated cells was lower than that in the untreated cells (Figure 6(a)). Because MMP-9 activity is a key regulator in the progression of bladder cancer [3, 12, 13], we investigated its regulatory mechanism in both nimbolide-treated bladder cancer cell lines. MMP-9 activity is regulated by the major transcriptional responsive elements, NF-*κ*B, AP-1, and Sp-1, which are common constituents present in the MMP-9 promoter region [11, 14, 15]. Therefore, nimbolide-mediated inhibition of transcriptional MMP-9 activity is investigated using EMSA experiment. As shown in Figure 6(b), binding activities of NF-*κ*B, AP-1, and Sp-1 were significantly inhibited in the presence of nimbolide in all the cells. These results clearly demonstrate that nimbolide inhibited MMP-9 activity by suppressing the binding activity of the transcription factors NF-*κ*B, AP-1, and Sp-1 in both bladder cancer EJ and 5637 cells.

## 4. Discussion

Many efforts have been continued to find agents with no or very low cytotoxic property that can suppress the growth and migration of malignant tumors. Natural products from the medicinal plants have been considered to be a safe and effective alternative in the prevention or treatment of cancer [24]. In a previous study, we found that nimbolide derivatives of neem seed and leaves selectively exert cytotoxic effects on cancer cells and induce apoptosis only in cancer cells [25]. The present study reports the exact molecular mechanism of nimbolide-induced inhibition of proliferation, migration, and invasion mediated by altered cell cycle regulation, specific signaling pathways, and transcription factor-associated MMP-9 expression in bladder cancer cells.

Nimbolide is a potent triterpenoid limonoid derived from the seed and leaves of neem trees. Our results indicated that nimbolide inhibited the proliferation of bladder cancer EJ cells in a dose-dependent manner at 12 h. Normally, cell growth and division are precisely regulated by cell cycle [8, 9]. Uncontrolled cell cycle regulation is one of the main hallmarks of tumor cells, which results in an abnormal proliferation of cells [8, 9]. Many types of anticancer agents have been shown to induce cell cycle arrest at a specific phase [26, 27]. Therefore, the control of proteins that regulate critical events of a certain cell cycle phase may be a useful strategy for anticancer therapy [27, 28]. A previous study demonstrated that nimbolide exerted a strong inhibitory effect on the growth of colon cancer cells owing to cell cycle arrest [29]. Flow cytometric analysis of U937 cells showed that nimbolide treatment resulted in cell cycle disruption by decreasing the number of cells in the G0/G1 phase [16]. Similarly, nimbolide was shown to induce G0/G1 phase in human cervical cancer, breast cancer, and glioblastoma cells [30–32]. The present results showed that nimbolide-induced inhibition of proliferation of both EJ and 5637 cells was owing to the induction of G2/M phase cell cycle arrest.

Under stressful conditions, two types of checkpoint kinases, such as ataxia telangiectasia mutated (ATM) and ataxia telangiectasia and Rad3-related (ATR) proteins, are activated, which leads to the induction of G2/M phase cell cycle arrest [9]. Chk1 can be phosphorylated at Ser345 residue by activated ATR, and Chk2 is believed to be activated via Thr68 phosphorylation in response to ATM [33, 34]. Two of the downstream proteins of Chk2 are Cdc25c and p21WAF1 that influence Cdc2 activity [8, 9]. Activation of Chk1 or Chk2 stimulates the phosphorylation of the cell cycle regulatory protein Cdc25c [9, 10]. G2/M transition is regulated by the formation of complexes between Cdc2 and cyclin B1 [9]. At the G2 phase progression, Cdc2/cyclin B1 complexes are inactivated in the presence of either Wee1 or Cdc2 phosphorylation at Tyr15 residue [9]. As cells enter into the M phase, dephosphorylation of Cdc2 at Tyr15 was caused by Cdc25c tyrosine phosphatases, which led to G2/M transition through Cdc2/cyclin B complex activation [9]. To investigate the regulatory mechanism of G2/M phase cell cycle arrest after nimbolide treatment, the expression level of regulators involved in the G2/M phase cell cycle was evaluated. Our immunoblot analysis demonstrated that nimbolide induced the phosphorylation of Chk2 in bladder cancer cells. However, the phosphorylation level of Chk1 was not altered in nimbolide-treated cells. In addition, the phosphorylation levels of both Cdc25c and Cdc2 increased after nimbolide treatment. Treatment with nimbolide decreased the protein levels of Cdc25c, Cdc2, and cyclin B1 and increased the expression of Wee1 and p21WAF1 in bladder cancer cells. These results suggest that nimbolide induced G2/M phase cell cycle arrest in bladder cancer cells via Chk2-Cdc25C-Cdc2/cyclin B1-Wee1 pathway and Chk2-p21WAF1-Cdc2/cyclin B1-Wee1 pathway. Taken together, the results of the present study indicate for the first time that Chk2 could be an inclusive target for the cell cycle-disrupting activity performed by nimbolide.

It has recently been demonstrated that the phosphorylation states of MAPKs (ERK1/2. JNK, and p38MAPK) and AKT are crucial events along with the dual signals regulating both cell growth and retardation of cellular activities [35–38]. It has recently been demonstrated that the phosphorylation of ERK1/2, JNK, p38MAPK, and AKT is closely linked with the inhibition of tumor cell proliferation in several tumor cell lines [37, 38]. Other studies have shown that the inhibition of tumor cell proliferation is involved in reduced phosphorylation of tumor cell-associated ERK1/2 and AKT [35, 36]. Our results indicated that nimbolide causes JNK phosphorylation, but not ERK1/2 phosphorylation, in bladder cancer cells. In addition, signaling of p38MAPK and AKT is downregulated in nimbolide-treated cells. Taking our previous results into account, it can be concluded that, along with cell types or culture condition, nimbolide may also affect different signaling pathways. The present results demonstrate that nimbolide-induced inhibition of proliferation of bladder cancer cells is mediated, at least in part, by increased JNK phosphorylation and impeded the phosphorylation signaling pathway of p38MAPK and AKT.

Metastasis is a complex series of processes in which tumor cells leave a primary site and migrate to different sites of the body through the circulatory system and establish malignant tumors [3, 39]. Migration and invasion of cancer cells are a critical step in cancer metastasis [3, 39]. In the present study, we observed that the migration and invasion of bladder cancer cells are inhibited by nimbolide treatment, thereby suggesting that nimbolide might be an effective molecule in the treatment of bladder cancer patients. MMPs, such as MMP-2 and MMP-9, consist of a multigene family of zinc-dependent ECM-remodeling endopeptidases that accelerates proteolytic degradation of ECM and thereby causes migration and invasion of cancer cells, which in turn results in the promotion of tumor metastasis [3, 11–15]. It is considered that MMP-9 is a crucial factor in the progression and development of bladder cancer [3, 12, 13]. Therefore, MMP-9 is amenable to therapeutic intervention by synthetic and natural inhibitors of bladder cancer; this provides perspectives for future studies [3, 12, 13, 40]. In the present study, nimbolide treatment of bladder cancer cells significantly reduced the expression of MMP-9. In agreement with our results, it has been reported that nimbolide disturbs migration, invasion, and MMP-2 and MMP-9 expressions in human breast cancer cells and pancreatic cells [41, 42]. NF-*κ*B, AP-1, and Sp-1 are transcription factors that regulate MMP-9 expression involved in the migration and invasion of tumor cells [11, 14, 15]. We found that nimbolide treatment reduced the activation of the transcription factors NF-*κ*B, AP-1, and Sp-1 in bladder cancer cells. The ability of nimbolide to suppress the transcription factor-associated MMP-9 expression suggests that it has a role in preventing migration and invasion of bladder cancer cells.

**Figure 7:**
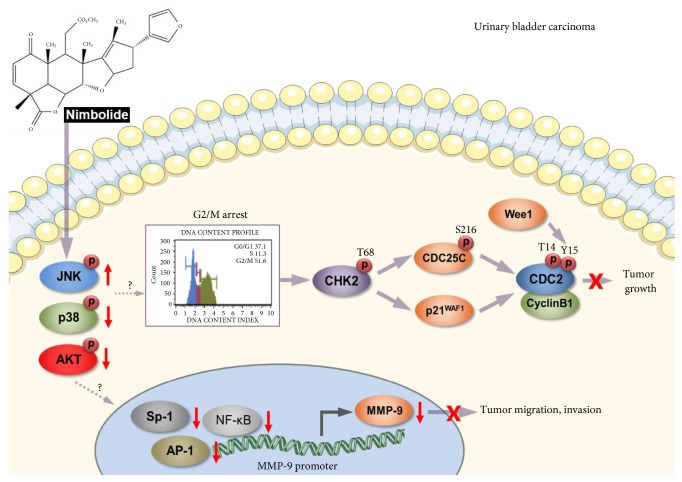
Schematic diagram depicting the proposed inhibitory mechanism of nimbolide in bladder cancer cells.

The nimbolide-induced antitumor effects on bladder cancer cells are summarized in Figure 7. The present study suggests that nimbolide inhibited the proliferation of bladder cancer cells through G2/M phase cell cycle arrest mediated by both the Chk2-Cdc25C-Cdc2/cyclin B1-Wee1 pathway and Chk2-p21WAF1-Cdc2/cyclin B1-Wee1 pathway. In addition, nimbolide increased JNK phosphorylation and reduced p38MAPK and AKT phosphorylation. Furthermore, nimbolide impaired the migration and invasion of bladder cancer cells by reducing MMP-9 expression, which was mediated by transcriptional suppression of the transcription factors NF-*κ*B, AP-1, and Sp-1. Therefore, nimbolide may have utility as a potential therapeutic target for bladder cancer treatment. Further* in vivo *studies are needed to evaluate the efficacy of nimbolide in the treatment of bladder cancer.

## 5. Conclusion

The utility of nimbolide, which is derived from the neem tree, was explored in bladder cancers. Our results indicate that nimbolide suppressed the proliferation of bladder cancer cells via Chk2-mediated G2/M phase cell cycle arrest. In addition, the antiproliferative effects of nimbolide in bladder cancer cells were associated with signaling pathway alterations. Furthermore, nimbolide-induced inhibition of migration and invasion in bladder cancer cells was caused by reduced MMP-9 expression, which was mediated by suppression of associated transcription factors. The present study demonstrated the precise molecular mechanisms underlying the chemotherapeutic effects of nimbolide on human bladder cancer cells. These results also provide valuable fundamental data for the development of a novel anticancer molecule.

## Data Availability

The data used to support the findings of this study are available from the corresponding author upon request.

## References

[1] Bray F., Ferlay J., Soerjomataram I., Siegel R. L., Torre L. A., Jemal A. (2018). Global cancer statistics 2018: GLOBOCAN estimates of incidence and mortality worldwide for 36 cancers in 185 countries. *CA: A Cancer Journal for Clinicians*.

[2] Sanli O., Dobruch J., Knowles M. A. (2017). Bladder cancer. *Nature Reviews Disease Primers*.

[3] Rovertson A. G., Kim J., AI-Ahmadie H. (2017). Comprehensive molecular characterization of muscle-invasive bladder cancer. *Cell*.

[4] Ghagane S. C., Puranik S. I., Kumbar V. M. (2017). In vitro antioxidant and anticancer activity of Leea indica leaf extracts on human prostate cancer cell lines. *Integrative Medicine Research*.

[5] Knowles M. A., Hurst C. D. (2015). Molecular biology of bladder cancer: new insights into pathogenesis and clinical diversity. *Nature Reviews Cancer*.

[6] Gerhardt D., Bertola G., Dietrich F. (2014). Boldine induces cell cycle arrest and apoptosis in T24 human bladder cancer cell line via regulation of ERK, AKT, and GSK-3*β*. *Urologic Oncology: Seminars and Original Investigations*.

[7] Mayer I. A., Arteaga C. L. (2016). The PI3K/AKT pathway as a target for cancer treatment. *Annual Review of Medicine*.

[8] Otto T., Sicinski P. (2017). Cell cycle proteins as promising targets in cancer therapy. *Nature Reviews Cancer*.

[9] Jeggo P. A., Pearl L. H., Carr A. M. (2016). DNA repair, genome stability and cancer: A historical perspective. *Nature Reviews Cancer*.

[10] Matheson C. J., Backos D. S., Reigan P. (2016). Targeting WEE1 Kinase in Cancer. *Trends in Pharmacological Sciences*.

[11] Lee S.-J., Cho S.-C., Lee E.-J. (2013). Interleukin-20 promotes migration of bladder cancer cells through extracellular signal-regulated kinase (erk)-mediated mmp-9 protein expression leading to nuclear factor (nf-kb) activation by inducing the up-regulation of p21waf1 protein expression. *The Journal of Biological Chemistry*.

[12] Bianco F. J., Gervasi D. C., Tiguert R. (1998). Matrix metalloproteinase-9 expression in bladder washes from bladder cancer patients predicts pathological stage and grade. *Clinical Cancer Research*.

[13] Davies B., Waxman J., Wasan H. (1993). Levels of matrix metalloproteases in bladder cancer correlate with tumor grade and invasion. *Cancer Research*.

[14] Bond M., Fabunmi R. P., Baker A. H., Newby A. C. (1998). Synergistic upregulation of metalloproteinase-9 by growth factors and inflammatory cytokines: an absolute requirement for transcription factor NF-*κ*B. *FEBS Letters*.

[15] Sato H., Seiki M. (1993). Regulatory mechanism of 92 kDa type IV collagenase gene expression which is associated with invasiveness of tumor cells. *Oncogene*.

[16] Gupta S. C., Prasad S., Tyagi A. K., Kunnumakkara A. B., Aggarwal B. B. (2017). Neem (Azadirachta indica): An indian traditional panacea with modern molecular basis. *Phytomedicine*.

[17] Gunadharini D. N., Elumalai P., Arunkumar R., Senthilkumar K., Arunakaran J. (2011). Induction of apoptosis and inhibition of PI3K/Akt pathway in PC-3 and LNCaP prostate cancer cells by ethanolic neem leaf extract. *Journal of Ethnopharmacology*.

[18] Elumalai P., Arunakaran J. (2014). Review on molecular and chemopreventive potential of nimbolide in cancer. *Genomics & Informatics*.

[19] Moga M., Bălan A., Anastasiu C., Dimienescu O., Neculoiu C., Gavriș C. (2018). An overview on the anticancer activity of azadirachta indica (Neem) in gynecological cancers. *International Journal of Molecular Sciences*.

[20] Pooladanda V., Thatikonda S., Bale S. (2014). Nimbolide protects against endotoxin-induced acute respiratory distress syndrome by inhibiting TNF-a mediated NF-*κ*B and HDAC-3 nuclear translocation. *Cell Death & Disease*.

[21] Bodduluru L. N., Kasala E. R., Thota N., Barua C. C., Sistla R. (2014). Chemopreventive and therapeutic effects of nimbolide in cancer: The underlying mechanisms. *Toxicology in Vitro*.

[22] Masters J. R., Hepburn P. J., Walker L. (1986). Tissue culture model of transitional cell carcinoma: Characterization of twenty-two human urothelial cell lines. *Cancer Research*.

[23] Capes-Davis A., Theodosopoulos G., Atkin I. (2010). Check your cultures! A list of cross-contaminated or misidentified cell lines. *International Journal of Cancer*.

[24] Li Y., Li S., Meng X., Gan R.-Y., Zhang J.-J., Li H.-B. (2017). Dietary natural products for prevention and treatment of breast cancer. *Nutrients*.

[25] Kashif M., Hwang Y., Hong G., Kim G. (2017). In vitro comparative cytotoxic effect of nimbolide: A limonoid from Azadirachta indica (neem tree) on cancer cell lines and normal cell lines through MTT assay. *Pakistan Journal of Pharmaceutical Sciences*.

[26] Lv T., Wang G. (2015). Antiproliferation potential of withaferin A on human osteosarcoma cells via the inhibition of G2/M checkpoint proteins. *Experimental and Therapeutic Medicine*.

[27] Ravishankar D., Rajora A. K., Greco F., Osborn H. M. I. (2013). Flavonoids as prospective compounds for anti-cancer therapy. *The International Journal of Biochemistry & Cell Biology*.

[28] Gordon E., Ravicz J., Liu S., Chawla S., Hall F. (2018). Cell cycle checkpoint control: The cyclin G1/Mdm2/p53 axis emerges as a strategic target for broad-spectrum cancer gene therapy - A review of molecular mechanisms for oncologists. *Molecular and Clinical Oncology*.

[29] Wang L., Phan D. D., Zhang J. (2016). Anticancer properties of nimbolide and pharmacokinetic considerations to accelerate its development. *Oncotarget*.

[30] Karkare S., Chhipa R. R., Anderson J. (2014). Direct inhibition of retinoblastoma phosphorylation by nimbolide causes cell cycle arrest and suppresses glioblastoma growth. *Clinical Cancer Research*.

[31] Elumalai P., Arunkumar R., Benson C. S., Sharmila G., Arunakaran J. (2014). Nimbolide inhibits IGF-I-mediated PI3K/Akt and MAPK signalling in human breast cancer cell lines (MCF-7 and MDA-MB-231). *Cell Biochemistry & Function*.

[32] Priyadarsini R. V., Murugan R. S., Sripriya P., Karunagaran D., Nagini S. (2010). The neem limonoids azadirachtin and nimbolide induce cell cycle arrest and mitochondria-mediated apoptosis in human cervical cancer (HeLa) cells. *Free Radical Research*.

[33] Rundle S., Bradbury A., Drew Y., Curtin N. J. (2001). Targeting the ATR-CHK1 axis in cancer therapy. *Cancers*.

[34] Manic G., Obrist F., Sistigu A., Vitale I. (2015). Trial Watch: Targeting ATM–CHK2 and ATR–CHK1 pathways for anticancer therapy. *Molecular and Cellular Oncology*.

[35] Zachos I., Konstantinopoulos P. A., Tzortzis V. (2010). Systemic therapy of metastatic bladder cancer in the molecular era: current status and future promise. *Expert Opinion on Investigational Drugs*.

[36] Zaravinos A., Lambrou G. I., Volanis D., Delakas D., Spandidos D. A. (2011). Spotlight on differentially expressed genes in urinary bladder cancer. *PLoS ONE*.

[37] Kamiyama M., Naguro I., Ichijo H. (2015). *In vivo* gene manipulation reveals the impact of stress-responsive MAPK pathways on tumor progression. *Cancer Science*.

[38] Vivanco I., Sawyers C. L. (2002). The phosphatidylinositol 3-kinase-AKT pathway in human cancer. *Nature Reviews Cancer*.

[39] Matrisian L. M. (1990). Metalloproteinases and their inhibitors in matrix remodeling. *Trends in Genetics*.

[40] Sier C. F. M., Casetta G., Verheijen J. H. (2000). Enhanced urinary gelatinase activities (matrix metalloproteinases 2 and 9) are associated with early-stage bladder carcinoma: A comparison with clinically used tumor markers. *Clinical Cancer Research*.

[41] Elumalai P., Brindha Mercy A., Arunkamar R. (2014). Nimbolide inhibits invasion and migration, and down-regulates uPAR chemokine gene expression, in two breast cancer cell lines. *Cell Proliferation*.

[42] Subramani R., Gonzalez E., Arumugam A. (2016). Nimbolide inhibits pancreatic cancer growth and metastasis through ROS-mediated apoptosis and inhibition of epithelial-to-mesenchymal transition. *Scientific Reports*.

